# Family Legacy of Diabetes-Related Behaviors: An Exploration of the Experiences of African American Parents and Adult Children

**DOI:** 10.1177/2333393619852343

**Published:** 2019-05-29

**Authors:** Brianna Routh, Tera Hurt, Donna Winham, Lorraine Lanningham-Foster

**Affiliations:** 1 Montana State University, Bozeman, Montana, USA; 2 Iowa State University, Ames, Iowa, USA

**Keywords:** diabetes, African American, intergenerational, high-risk families, nutrition

## Abstract

African Americans are at higher risk of developing type 2 diabetes mellitus (T2DM), and this risk may be influenced by familial experiences and cultural norms throughout the life course. This led us to conduct this study of 20 African American families with strong histories of T2DM to explore familial complexities that prevent or help manage diabetic symptoms. Experiences were analyzed inductively through individual family profiles created using content-analytic summaries. When profiles were further analyzed for emerging and theoretically informed data patterns, two themes emerged: (a) family interactions characterized by T2DM-related actions and communication patterns, and (b) intergenerational patterns of openness characterized by variations in approach within generational cohort and parental gender. Through inquiries related to intergenerational experiences with T2DM, nursing and health care professionals may be better able to tailor and promote success for prevention and management of behaviors among high-risk African Americans.

## Introduction

Type 2 diabetes mellitus (T2DM) is a leading cause of morbidity and mortality, particularly for non-Hispanic African American adults in the United States (Centre for Disease Control and Prevention, 2017). According to the Iowa Department of Public Health, which tracks these trends in the state where this study was conducted, the prevalence of people diagnosed with T2DM increased from 3.8% in 1991 to 7.5% in 2010, with the risk of having T2DM for African American adults more than twice that of Whites (13% for African American women and men vs. 6% for White men and women) (Muldoon, 2011). Despite the apparent greater risk of T2DM, African Americans were less likely than their White counterparts to report feeling as though they were at risk of T2DM (Gallivan, Brown, Greenberg, & Clark, 2009). Family history across generations, independent of race, body mass index, age, or income, is one of the strongest predictors of obesity and T2DM (Drong, Lindgren, & McCarthy, 2012; Valdez, Yoon, Liu, & Khoury, 2007). With only limited evidence suggesting genetic T2DM transmission, it is increasingly important to understand the role of the environment, specifically the familial environment, in contributing to development of T2DM (Drong et al., 2012).

Within African American culture, collectivism, interdependence, and cooperation are valued, and family definitions are often flexible. The term “Family” often includes not only persons linked by marriage, blood, and adoption but also persons who are frequently and substantially involved in one’s life (Peyrot et al., 2015). As family members, particularly parents, are often considered to be key gatekeepers to early nutrition behaviors (Maher, Fraser, & Wright, 2010), it is important to consider the role of family ties because T2DM affects families, not just individuals (Mayberry & Osborn, 2012; Rosland, Heisler, & Piette, 2012). Specifically, familial interactions and experiences with T2DM may influence individuals’ preventive and management behaviors throughout their life course. We, therefore, conducted the present study of 20 African American families exhibiting strong histories of T2DM to explore complexities that may contribute to behaviors that can prevent or help manage diabetic symptoms.

### Theoretical Framework

To understand how and why family member interactions may impact individual T2DM-related behaviors, this analysis was informed by Bowen’s Family Systems Theory (FST) and Bandura’s Social Cognitive Theory (SCT). FST posits that family members are interconnected and interdependent, with each person affecting others within family networks (Bowen & Devine, 2011; Kerr, 2002). FST also suggests that through relationships and intergenerational transmission, family can have potentially long-lasting influences on behaviors (Kerr, 2002). Similarly, environmental influences over time, such as family and cultural contexts, are a key tenet of the SCT (Bandura, 1986, 2012). SCT expands on Bandura’s earlier Social Learning Theory (SLT) that identified the importance of past experiences through observational learning and reinforcement, and further suggested that such experiences may not only shape learning through behavioral capabilities, such as developing knowledge and skills, but also may influence an individual’s expectations and self-efficacy through enacting specific behaviors (Bandura, 2012).

### Factors Influencing Behaviors

Despite the vast body of research on T2DM, most studies to date have focused only on contemporaneous factors related to T2DM management, without attending to the potential learning that can occur between an individual and his or her familial environment over time. We refer to this as the multigenerational legacy of T2DM. Multiple familial interactions related to the ritual of meals and food consumption have been associated with eating behaviors and obesity outcomes. Parenting strategies such as repeated food exposures and positive facial expressions have also been shown to increase consumption among children of fruits, vegetables, or novel food (Anzman-Frasca, Savage, Marini, Fisher, & Birch, 2012; Barthomeuf, Droit, Volet, & Rousset, 2012; Cruwys, Bevelander, & Hermans, 2015). Alternatively, strategies that restrict some foods, use food as a reward, or provide inadequate structure are associated with higher obesity risk for children (Johnson, Welk, Saint-Maurice, & Ihmels, 2012; Skouteris et al., 2011). Some research studies support authoritative parenting and modeling practices as potentially effective obesity-prevention strategies among African American adolescents (Schneider, Wilson, Kitzman-Ulrich, George, & Alia, 2013). Although more research is needed in this area, experts suggest that strategies used in nutrition interventions (e.g., education and promotion of self-awareness), may similarly promote healthy food behaviors shared among family members (Larsen et al., 2015). Despite extensive research regarding specific food-parenting behaviors, variations in such practices might differently influence T2DM-related nutrition-based behaviors in the home (Musher-Eizenman & Kiefner, 2013). Consistent with the SCT, such familial experiences may prompt beliefs, knowledge, or skills that individuals can incorporate into their learning and T2DM-related behaviors across their entire life spans.

#### Cultural norms

Families can serve as a significant source of information about cultural beliefs and observational learning related to health and illness (Scollan-Koliopoulos, O’Connell, & Walker, 2007). Although many T2DM risk factors have been associated with race, a study of cultural characteristics may be even more influential with respect to understanding T2DM risk and prevention (Scollan-Koliopoulos, Rapp, & Bleich, 2012). Cultural acceptance of larger body size or an optimistic bias may limit personal perceived risk (Barroso, Peters, Johnson, Kelder, & Jefferson, 2010; Doolen, Alpert, & Miller, 2009; Parham-Payne, 2013), and the medical system is often viewed skeptically among some African Americans as impractical and culturally irrelevant (Airhihenbuwa et al., 1996; Bhattacharya, 2012). Alternatively, strong religious beliefs and spirituality have often been associated with better self-care practices among African American women (Watkins, Quinn, Ruggiero, Quinn, & Choi, 2013). Taken together, such research supports the importance of beliefs and cultural norms in many African American communities, and this may impact perceptions of T2DM or obesity risk for their children (Barroso et al., 2010; Doolen et al., 2009).

In addition, as with many cultures, food plays a central role in many African American families (Vanstone et al., 2013). Overweight adolescent African American girls expressed the view that family and cultural perceptions related to eating “grandma’s cooking” outweighed considerations of health (Boyington et al., 2008). Adult African Americans in the southwest United States expressed the view that modifying traditional foods for better health would not be acceptable due to loss of flavor in the original dishes. Although many knew which foods were healthier, they stated their food choices were based on convenience and economic concerns (Der Ananian, Winham, Thompson, & Tisue, 2018).

Although many studies support the importance of African American cultural identity on food choices, one study suggested that older generations may regard this cultural identity as having had more influence on their food choices than it did for younger generations (Airhihenbuwa et al., 1996). Some older African American women felt that education should be directed to the younger generations where it could be more beneficial in terms of prevention and behavior change (Der Ananian et al., 2018). Further research is needed to understand how cultural context can impact the transmission of T2DM-related behaviors between generations among families already at high-risk of T2DM.

#### Family patterns

Each family subsystem is unique, and so there may be variations within families that impact T2DM-related behaviors. Although multiple researchers have found strong similarities between siblings with respect to food preferences (Pliner & Pelchat, 1986; Skinner et al., 1998), additional research suggests siblings may exhibit a variety of obesity-risk-related behaviors. Specifically, parents may have unique interactions with each of their children depending on a child’s weight status, resulting in potentially different parent–child food influences (Berge, Tate, Trofholz, Conger, & Neumark-Sztainer, 2016). Among African American individuals, family members can serve as supporting elements with respect to T2DM self-care, but perception of them as hindrances may also influence a patient’s self-efficacy toward care (Mayberry & Osborn, 2012). Only limited research has explored exactly which family variations tend to exhibit stronger associations with preventive T2DM practices among African American families with strong family histories of T2DM.

In addition to cultural influences, family patterns over time may also be particularly important in shaping T2DM-related behaviors. Specifically, a family history of T2DM has often been associated with increased awareness of risk and preventive behaviors for African American adults (Omolafe, Mouttapa, McMahan, & Tanjasri, 2010). Supporting FST suggestions related to multigenerational influences, Scollan-Koliopoulos et al. (2007) found associations between recollections of familial experiences with T2DM and subsequent perceptions or T2DM-related behaviors. One study indicated that, even with T2DM education programming, learned familial patterns may still shape behaviors among African American adults (Scollan-Koliopoulos, Walker, & Rapp, 2011). We will seek further understanding of the potential influences of family interactions over time by exploring reported experiences of parents and adult child dyads within African American families with T2DM.

This study explored qualitative data pertaining to existence of a T2DM-related nutrition legacy across generations. The context focused on experiences of a parent with T2DM and one of their adult children to explore how such observations of experience translated into current nutrition behaviors. We explored reports of familial experiences pertaining to nutrition ranging from direct interactions to indirect observations, and particularly on past T2DM-related experiences of these high-risk families. As both SCT and FST would suggest that such reported experiences informed participants’ perceived family nutrition legacies and potentially shaped their current T2DM management and prevention behaviors, we sought to investigate the following questions from the perspective of African American parents and adult children: (a) How did participants report family influences as it concerned T2DM and nutrition? and (b) What similarities and differences were there in nutrition and T2DM-related familial interactions across generations? To our knowledge, this is the first study of its kind to conduct interviews with African American parent–adult child dyads to explore these issues.

## Methods

### Sample

This study recruited a subsample of families from Wave 6 of the longitudinal study Family and Community Health Study (FACHS) that has followed more than 800 African American primary caregivers and their 10- to 12-year-old children residing in Iowa and Georgia since 1997 (Cutrona et al., 2003). FACHS participants consented to be contacted for further research opportunities. In 2014, using principles of purposive sampling, a subsample of 57 families was invited to this project. In the Des Moines sample (*n* = 41), 12 were ineligible, 7 were unable to reach, 6 declined (sample reasons: life is hectic, adult child did not reside in Iowa nor planned to return home within project period), and 1 was a no show for the interview. Of the 41 families, 15 parent–adult child dyads enrolled in the study and completed the interview. In one family, both the parent and their romantic partner were diagnosed with T2DM. As this project was descriptive and exploratory in nature, the study investigators chose to include these two participants and both adults took part in the interview with the adult child. Among the Waterloo sample, letters were mailed to 16 families. Of them, 6 were unable to reach, 2 declined, 2 were ineligible, and 1 parent died. Five families residing in Waterloo, Iowa took part in the project. In total, 20 families (21 parents and 20 adult children; 14 males and 27 females) met the eligibility requirements and agreed to complete the research study (35% retention rate).

All invited families resided either in Des Moines or Waterloo, Iowa, and a parent had previously reported having T2DM. To ensure that the subsample further reflected a strong family history of T2DM, as defined by Valdez et al. (2007), to be eligible for the study, (a) families had at least one parent participant who was diagnosed with T2DM; (b) this parent had at least one other blood/biological relative (i.e., grandparent, parent, brother, and sister), now living or deceased, who was also diagnosed with T2DM; and (c) this parent had an adult child both available and willing to participate in the interview. We describe the sample in Table 1.

**Table 1.** Sample Characteristics (*n* = 21).

Gender	Male	Female
Parents	6	15
Adult children	9	10
Age	Mean	Range
Parents	56	46–78
Adult children	27	20–28
Income	Mean annual earnings	Range
Parents	US$30,000–US$39,999	Less than US$4,999–Greater than US$70,000
Adult children^a^	US$30,000–US$39,999	Less than US$4,999–Greater than US$70,000
Education	Mean education	Range
Parents	Some college/technical school	Less than High school to college degree
Adult children	Some college/technical school	Less than High school to college degree
Diagnosed with T2DM	Males	Females
Parents	6	15
Adult children	0	1

*Note.* T2DM = type 2 diabetes mellitus.

a One participant did not respond.

### Procedures

Working within an interpretivist paradigm that suggested that this phenomenon could be best understood from the subjective experiences of multiple family members, we focused on understanding how parents and adult children perceived their personal experiences related to T2DM and nutrition (Creswell, Plano Clark, Gutmann, & Hanson, 2003). A semi-structured style of open-ended questioning was used to collect both parents’ and adult children’s perspectives, and the PI/SA (principal investigator/second author) was flexible in her line of questioning, rephrasing or following-up on questions as needed to ensure clear understanding of participants’ perspectives.

Participants were invited into the study through a letter and a subsequent phone call, during which interested participants were screened for eligibility. Data were collected from May 2014 through August 2014. Interviews were conducted by the PI/SA. The PI/SA was a female faculty member with a doctorate and more than 20 years of experience conducting qualitative studies. She shared the same racial background as the participants. The PI/SA did not establish a relationship with the participants prior the study, and in almost all cases, it was her first time meeting the study participants. The participants were advised of the PI/SA’s name, university position, and reasons for doing the research, consistent with the information provided in the informed consent. As the interview unfolded, the PI/SA shared personal connections to the study topic, where relevant.

Graduate student assistants helped participants with completion of informed consent documents and demographic surveys, checked the completion of study materials, distributed and collected materials, and followed up on emergent insights during the qualitative interview. That is, in addition to the PI/SA, they were present for the interview with the respondents. To maximize participants’ levels of comfort, the interview team informally conversed with participants about the study and key events in their lives in an attempt to build rapport and connect with them during the interview process. Parents and their adult children completed one interview in which they were simultaneously interviewed. Most interviews lasted approximately 2.0 hr and most were conducted in a private room at the local Boys and Girls Club. For one family with a homebound participant, we conducted the interview in his living room, per his request. Each participant was paid $70 for completing the interview. This study was approved by the Iowa State University Institutional Review Board, and informed consent was secured from all parents and adult children at the time of data collection.

In general, each interview focused on nine broad areas—first experiences or recollections of T2DM and T2DM socialization within the family; diabetic diagnosis; meaning of being diagnosed with T2DM; resources (e.g., individual, family, social, medical, and economic) for helping manage T2DM; food; coping resources; exercise; adverse events that could exacerbate T2DM; and family support and intervention programming. We focused on data describing the experiences of intergenerational transmission of nutritional information, skills, and understanding. Questions included “What kind of foods do you eat? Who prepares the food you eat? What are the family traditions relative to food?” After each interview concluded, the PI/SA and graduate research assistants recorded observational notes by hand and stored these documents in the participants’ files. Data saturation was not achieved, but rather, the study ended when grant funding could no longer support recruiting any additional families to the project.

All qualitative interviews were documented using digital recorders and stored after assigning participant identification numbers to ensure confidentiality. Recordings were transcribed by an experienced transcriptionist to document the spoken word (Miles, Huberman, & Saldana, 2013). Undergraduate and graduate research assistants checked the recordings against the transcripts for accuracy (Carlson, 2010), and inconsistencies were corrected using red font to identify the correction in the written record. Transcripts were not returned to participants for comment or correction. Transcripts were available both in Word and MAXQDA, a qualitative data management and analysis program. Qualitative data were independently collected and results were compared at the analysis stage (Terrell, 2012). In addition, quantitative demographics collected in the interview were entered into an Excel spreadsheet for analysis.

### Analyses

To inductively capture parental and adult children’s meanings of T2DM, we performed content-analysis to identify themes in the qualitative data collected (Creswell et al., 2003). The four authors met to discuss the manuscript and collaborate on data analysis using the following procedures. First, the second author reviewed the transcripts and wrote analytic memos for tracking both initial and continuing observations (Kuckartz, 2014). The authors reviewed transcribed data, and after thorough review, codes were assigned to sections of text referring to potential T2DM-related intergenerational connections (Kuckartz, 2014). Codes were derived theoretically from the SLT and SCT frameworks. Specifically, SLT tenets of direct or indirect modeling, verbal instruction, and emotional influence were used to theoretically group familial intergenerational interactions. Additional emergent codes for more fully capturing intergenerational connections (Kuckartz, 2014) were identified and refined by the research team and assigned during a second round of data coding.

Demographic information included sex and age of both parent and child. Family profiles were then used to create a content-analytic summary of each family based on a brief review of parent and adult child codes and demographic information (Kuckartz, 2014; Saldaña, 2015). Specifically, Miles, Huberman, and Saldana (2014) defined a content-analytic summary table as “a matrix display that batches or brings together all related or pertinent data from multiple cases into a single form for initial or exploratory analysis. Each cell of the matrix specifies the dimensions you are interested in” (p. 148). This process reflects data selection and condensation. The research team explored similarities and differences among intergenerational family profiles to reach consensus on emerging patterns (Kuckartz, 2014; Ojeda, Flores, Meza, & Morales, 2011; Wolcott, 1994). Study participants did not provide feedback on these findings.

The first and second authors first familiarized themselves with the qualitative data by reviewing the transcripts to seek understanding of participants’ responses, then performed data reduction to more sharply focus on important aspects of the participants’ experiences most relative to nutrition. After reviewing both parents’ and adult children’s experiences in the family profiles table, the authors compared data across cases to explore similarities and differences in the nutritional legacies, a process consistent with a cross-case analytic approach (Miles et al., 2014). The analytical procedures used involved iterative sequences of reviewing, categorizing, verifying, and drawing conclusions from the data (Miles et al., 2014).

At each stage of analysis, the first and second authors reviewed and modified the codes until a consensus with respect to uniformly understanding participants’ experiences was reached. Authors compared findings from their independent analyses and then discussed data themes in a collaborative way (Saldaña, 2015). The importance of each author recording her own work cannot be overstated; this helps in reducing difficulties attributed to dissimilarities in the way the four authors analyzed the data (Saldaña, 2015). All authors ultimately agreed with the themes that emerged from the data, and there were no subsequent unresolved disputes about the data analysis. In summary, this analytic process demonstrated dependability in data coding, similar to reliability in the quantitative paradigm (Anfara, Brown, & Mangione, 2002).

## Results

Two main themes emerged from content analysis of the multigenerational family profiles. With respect to research question 1 (i.e., How did participants report family influences as it concerned T2DM and nutrition?), we noticed themes and subthemes aligning with the SCT and related to types of familial interaction, including direct interaction, observed interaction, and communication (awareness and instruction). With respect to research question 2 (i.e., What similarities and differences were there in nutrition and T2DM-related familial interactions across generations?), there were primary themes of openness related to T2DM in familial interactions with subthemes of generational cohort and gender (Table 2).

**Table 2.** Summary of Themes.

Themes	Subthemes
Theme 1. Family Interactions	Actions: “Lead by example.”
	Communication: “My sister talks about it all the time, just what I can’t eat, what I shouldn’t drink.”
Theme 2. Intergenerational Patterns of Openness	Generational cohort: “It was hush-hush.”
	Parental gender: “she used to come over and watch what I eat.”

### Family Interactions

To better understand how families influenced their members’ knowledge about T2DM and how nutritional information was transferred across generations, we explored family interactions. Participants discussed specific types of T2DM-related familial interactions experienced throughout their lives, including direct and indirect actions or communication. Although many participants did not recall singular incidents they had experienced with a family member, they often expressed broad statements about familial experiences. We outlined such interactions with representative quotes used to illustrate the participants’ experiences.

Familial interactions that shaped T2DM-related behaviors included experiences with T2DM management behaviors, potential prevention-related behaviors, and resulting health complications. Although both management behaviors and health consequences were reported through all types of familial experiences, ranging from communicating awareness to observed interactions, preventive behaviors were largely described using verbal communication. Alternatively, less preventive behaviors or “bad habits” were more often described in direct or observed interactions that occurred within a family. Some participants explicitly stated that they did not feel anything was transmitted from previous generations either about T2DM or T2DM-related behaviors and nutrition. Although those participants often did not share communication or direct interactions, they were usually able to identify other observed familial experiences.

#### Actions

Participants recalled both direct and indirect interactions with family members. Participants described situations in which a parent or family member was modeling T2DM risk-reducing strategies for a younger generation. Other participants identified examples in which a family member engaged them in prevention strategies such as learning to cook with different ingredients or using healthier cooking practices (e.g., baking vs. frying). One adult daughter described how she had developed a legacy through actions, though her family rarely discussed T2DM. She recalled, “You get used to eating a certain way for so long and pass down recipes and stuff like that.” Some participants also noted that knowledge passed on to them reinforced what they considered to be bad habits, particularly with respect to food choices and food preparation techniques. Multiple participants described taking action because they didn’t want T2DM for themselves, for their children, or for their grandchildren. One grandmother who was diagnosed with T2DM expressed why she “lead(s) by example.” She said,

I know that’s why all the grandkids know I’m a diabetic. And they know the basics of what diabetes is, kind of. That something in my body doesn’t work right, so I have to be careful of eating too much sugar cause my body won’t process it. It can make me sick. They knew that I lost my vision. You know, so they watch me check my blood. They see all that. They don’t like it, so they are conscious that if they don’t take care of their self, that’s how they could, you know, end up. Not in a scary way but in a conscious way that you know you got to take care of yourself and eat healthy.

#### Communication

Participants also described T2DM experiences involving communication between family members, most of which was perceived as supportive or instructive in nature. At times, these communications raised awareness about T2DM-related nutrition issues, whereas other experiences described could be classified as prescriptive in nature. Often, these two types of verbal communication occurred in tandem in the form of awareness followed by suggested behaviors. One adult daughter described raising her parent’s awareness regarding high blood sugars and suggested behaviors to prevent further challenges:

Like sometimes I’m like, “Oh, you know, like your sugar is high. Why don’t you exercise?” Or like I’ll tell her, “I mean you don’t need any more regular Pepsi. You had three today. You had enough today.”

When describing communication that raised awareness, participants’ statements were often related to the prevalence and risk of T2DM. Similar to leading by example, participants described sharing personal testaments of their experiences with T2DM as raising their awareness. In addition, many within the parent generation described growing up and hearing about “the sugars,” which led many to believe sugar intake was the cause of T2DM. Even though many recognized that there was more to T2DM than just simple sugar intake, many still related their experiences, preventive efforts, and beliefs to using sugar, often meaning that carbohydrates, fats, or fibrous foods were not discussed.

In addition, participants described how family members communicated prescriptions for how they might manage T2DM. Some of this advice was more general, usually described as eating right, whereas others recalled suggested nutritional behaviors from family members, such as specific foods to avoid. For example, one mother with T2DM described that she appreciated her sister’s advice: “My sister talks about it all the time, just what I can’t eat, what I shouldn’t drink and no sweet tea and stuff so she reminds me constantly what it is. Which is good, I mean, I like it.” Others described more general recommendations to “eat healthy.”

### Intergenerational Patterns of Openness

Through actions, observations, and communication, participants reported that previous generations had affected their personal understanding and T2DM behaviors. In alignment with the SCT, because T2DM nutrition care behaviors were shaped by complex factors and experiences over time (Ali et al., 2013), varying familial influences could lead to differences in whether or not risky T2DM behaviors were transmitted across generations. To understand how shared experiences played out within families across generations, we compared experiences among parents diagnosed with T2DM and adult children within the same family.

When examining experiences shared by adult children and parents diagnosed with T2DM, we found that almost all participants recalled T2DM-related familial interactions, and they took these descriptions even further by indicating that they often felt that such experiences encouraged or supported them in making better choices. Some participants felt they had grown past some of the habits they viewed as bad from their childhood. One mother diagnosed with T2DM described how she had seen the good and bad, and she “learned to try to start eating smaller portions” and was taken off “diabetic pills” due to stable A1C levels. Alternatively, some of these “bad” habits were still present in both parent and adult children’s lives. These habits were learned from their families in childhood, with one adult son stating, “I mean it’s just greasy growing up and you just catch on to the bad habits or you have the bad habits you know.” Another father who was diagnosed with T2DM lamented the potential for bad habits to persist across generations. He said, “Make sure that you don’t let tradition fall into your eating habits.” Although types of interactions as well as what they were interacting about varied across both parents diagnosed with T2DM and adult children’s descriptions, there were some similarities and differences between what pairs noted based on reporting participant gender and which generational cohort they were in. The overarching theme of open communication emerged when comparing familial experiences around T2DM for both generational cohort and gender.

#### Generational cohort

One father with T2DM described observing family members with T2DM complications, knowing that this was not something to be discussed. He recalled, “It was hush-hush.” He described discussing T2DM with his uncle in this way: “You know you got diabetes. What is diabetes, sugar? He replied, ‘Leave me alone’. I said, ‘Okay’.” Alternatively, his own daughter described feeling T2DM was more openly discussed in her childhood:

My dad never complained or whined about it. [He] just did what [he] had to do so I guess that made me less fearful if I was to get it, you know, life goes on. You have diabetes, but life goes on just you got to take care of yourself and watch.

Participants perceived openness toward T2DM differently in their T2DM-related childhood experiences. Descriptions of childhood experiences by both parents diagnosed with T2DM and their adult children tended to reflect an increasing perception of openness of familial interactions related to T2DM with each subsequent generation.

Consistent with previous research, multiple parents and adult children with a family history of T2DM seemed to understand that their children or grandchildren were at higher T2DM risk, wanting to ensure they were aware of and educated about their risk of T2DM and how to prevent it (Nsiah-Kumi, Ariza, Mikhail, Feinglass, & Binns, 2009). Although a few parents expressed skepticism about the likelihood of future generations making changes toward healthier food choices, there were others who seemed more optimistic and ready to help future generations learn about T2DM. In some cases, parents who may have felt they did not do enough to educate their own children stressed the importance of providing grandchildren with information and suggested behaviors about T2DM. Many of the adult children shared examples of how they were connecting concepts, behaviors, and impressions taken from previous generations to the ways they were trying to raise their own children and model preventive measures for them. One adult child of a mother diagnosed with T2DM described how she was moving beyond the view of T2DM as just a function of heredity. She said,

Sometimes when my mom talks about it being like hereditary, it’s like I would like to say, you know, by me trying to do preventive measures, that it can be stopped and it can be corrected. I mean, maybe I’ll have to deal with the symptoms, but I can change it with my diet and exercise and stuff to where I’m not trying to take all that medicine and stuff.

Parents with T2DM described how they recognized the need to take preventive action for themselves or their families when their children were young, whereas others took longer to make these connections and shared that they began making lifestyle changes later in life. Still others did not describe any lifestyle changes or saw no need to adjust their life and nutritional practices because of T2DM.

#### Parental gender

Although there were counter-examples, participants in general reported less open communication with fathers than with mothers. Alternatively, adult children did report that they had experiences through observation of family members. This suggested a similar pattern, although with a less extreme description for all but one adult child, to the experiences parents diagnosed with T2DM described when they felt T2DM was a taboo topic in their childhood homes. Mothers were more likely to engage in open communication and intentional modeling with daughters; there were cases in which this occurred and successfully promoted less risky behaviors. One adult daughter explained how her grandmother frequently communicated and educated both her and her mother about T2DM-related management:

My grandmother, she exercises a lot and she watches what she eats and everything like that. And when I first got it (T2DM) she used to come over and watch what I ate, make sure I exercised and make sure I was taking my blood sugar and stuff like that. She does the same for my mom as well.

There were several notable counter-examples by gender. The father who experienced the “hush hush” childhood legacy said that he wanted to create a different legacy for his own children. His daughter recalled being actively involved in her father’s T2DM management, including diet and preventive practices. Both generations had become advocates for education and prevention in future generations. In another family, a mother with T2DM did not recall learning much about T2DM from her family, leading her to actively avoid sharing interactions with her children around T2DM, such as going into another room to give herself insulin shots, in the hope that her children would not be afraid or see T2DM as a death sentence. Her two children were present for the interview and shared limited understanding of T2DM care and potential implications; her adult daughter was there in a support/caregiving capacity, whereas the other adult child, a son, met the eligibility guidelines for the study. Although limited research has suggested vinegar may lower postprandial glucose levels among healthy subjects (Östman, Granfeldt, Persson, & Björck, 2005), her son shared the broader misconception that vinegar might cure T2DM. The only belief the daughter expressed about T2DM was that, without care, one could lose body parts.

## Discussion

This study sought to answer these two research questions: (a) How did participants report family influences as it concerned T2DM and nutrition? and (b) What similarities and differences were there in nutrition and T2DM-related familial interactions across generations? We explored contributing personal and familial factors and patterns associated with the legacy of T2DM for African American families with strong histories of this chronic disease. Among the findings were that multiple types of interactions, both intentional and unintentional, occurred in African American families with strong histories of T2DM, and that these interactions were related to multiple stages of T2DM care or prevention. The findings emphasized the importance of personal beliefs and emotions about T2DM, how these factors shape family interactions, and how they were later viewed. Patterns were identified related to how parents interacted with children across generations and genders, with the common present-day theme of wanting future generations to be more informed about T2DM.

### Previous Experiences Inform Current Behaviors

From this subsample of families with strong histories of T2DM, both parents diagnosed with T2DM and adult children described family experiences and beliefs related to T2DM risk and management in alignment with the SCT. Although a variety of interactions were described, most of the observations occurred unintentionally, whereas direct interactions and communication were often intentional, efforts to transfer information, educate, and build skills from older to younger generations. As participants recalled experiences, often from childhood, as a part of their family’s legacy of T2DM, these interactions fueled perceptions, beliefs, and subsequent behaviors (Scollan-Koliopoulos et al., 2007). Participants also described varying levels of both acknowledgment and denial of T2DM in their familial interactions, similar to findings describing exploration of individual perceptions among older adult African Americans managing T2DM (Spruill, Magwood, Nemeth, & Williams, 2015). These findings supported the literature that suggested that experiences and observations throughout life influenced understanding, skill-sets, and learning, that, in turn, could lead to acceptance, nutrition behavior maintenance, or change (Sahoo et al., 2015).

Consistent with findings from other studies on multigenerational transmission related to illness and the FST, one family member’s beliefs may influence other family members’ beliefs and actions (Scollan-Koliopoulos et al., 2007). Participants identified influential familial connections related to nonfamily experiences, emotions, and beliefs. Not only did the experience matter, but the emotions it invoked or the beliefs it confirmed or dissuaded fueled how behaviors developed for participants. It is crucial for practitioners to not only understand such experiences but also consider the associations drawn that could influence a patient’s learning or behavioral change.

### Relationships Within Families Inform Behaviors

For African American families with strong histories of T2DM, gender roles and norms as well as general communication openness between parent and child may prompted unique T2DM-related family legacies. Consistent with a growing body of research, we found that variations in gender roles among African American men and women in providing support and coping with the stress of household nutrition-related behaviors and T2DM management (Seawell, Hurt, & Shirley, 2016). Similarly, research in rural Appalachian families with T2DM in which women were found to more fully engage in supportive practices than men, potentially based on their family role of caring for others (Denham, Manoogian, & Schuster, 2007). Individual gender differences in health behaviors contributed to the parent–child dyadic health interaction literature as well. A meta-analysis of parent–child physical activity literature indicated positive associations in both same and opposite sex dyads and child physical activity, but parental and maternal support was particularly important influence on girls’ activity (Yao & Rhodes, 2015). Although Yao and Rhodes (2015) found no differences in communication behaviors among mother–child dyads regardless of the sex of the child, the current findings suggested influences of gender roles and norms may influences support behaviors. Specifically, gender norms and roles within families may contribute to variation in communication openness, in relation to T2DM and sensitive health topics for these African American families.

From other parent–child literature, we know that open communication on sensitive topics can at times serve as a support or buffer to negative effects with respect to associated health outcomes. Although there are relatively little data exploring the impact of parent–adult child communication openness on health, there is a body of research examining mother–adolescent communication, particularly with respect to sensitive topics such as sex. From that body of literature, we know that mothers communicate differently with sons and daughters, so these findings support existence of differential communication strategies between parent–child gender dyads (Kapungu et al., 2010). Similarly, there may be cultural and gender-related influences on just how open discussions of T2DM are within and between generations. Regardless, previous research indicates that there is at least a perceived stigma or taboo for some African American families in talking about T2DM (Boone & Lefkowitz, 2007). Although this stigma appears to be less prominent in the current parent–adult child dyadic relationship, it has been described as a prominent part of childhood experiences for the older generation (Noakes, 2010).

### Generational Similarities and Differences Inform Behaviors

There were also trends of cohort differences between parents and adult children’s eating patterns related to T2DM prevention. Variation in eating patterns may be related to larger transformation of the food system, indicating potential variations in environmental influences as described by the SCT (Bandura, 2012). More specifically, African American culture and beliefs about which foods help prevent or manage T2DM and how family members should interact around health may have shifted overtime. These shifts in how individuals interact with and perceive their food system may contribute to differentiation seen in the multigenerational transmission process described by the FST (Kerr, 2002). Exploring these connections between environment and family transmission with African American families with strong histories of T2DM may help practitioners identify potential points of difficulty or success in prevention and management strategies.

In addition, and important in the future for addressing families with strong histories of T2DM, our findings indicate that current generations, both with and without T2DM, feel the need to raise awareness and prevent T2DM for future generations. In alignment with the FST, these adult generations are acknowledging and considering the impacts of multigenerational transmission of beliefs and behaviors. Care for the future may serve as an incentive to facilitate family-level changes in behaviors that can benefit grandparent, parent, and child. Alternatively, not all parents made these changes for their own children, and many were still not following all recommended practices at the time of interview. As multiple researchers who have studied individuals with strong family histories of T2DM have suggested, avoidance or complacency in enacting preventive or management behaviors may be due to personal fatalism regarding T2DM due to family and community history of diagnosis (Pyatak, Florindez, Peters, & Weigensberg, 2014; Scollan-Koliopoulos et al., 2007). Future research should explore how both acknowledgment of T2DM risk and hope for the future can influence behavioral change for both the individual and the family unit.

It is important to note that this study’s findings have some limitations. First, this was an exploratory study, so even though we were able to get a reasonable dispersion of mothers/fathers and adult daughters/sons, we still had a relatively small sample size from which to draw our conclusions. Second, the study was cross-sectional in nature, so we were not able to test direct effects of family member relationships, interactions, or beliefs. Alternatively, many of these findings were retrospective in nature, allowing us to understand the most salient and perceived as important experiences. The use of qualitative data allows us to illuminate the voices and experiences of families with strong histories of T2DM that are often overlooked or lumped together with other segments of the population. These findings point to a need for further analysis on this topic.

## Implications

This study has implications, for current health professionals as well as for future studies, to inform T2DM prevention and management for African American individuals with strong family histories of T2DM. Many scholars have underscored the importance of family support in helping improve T2DM prevention and management through cues to action, direct assistance, reinforcement, and knowledge (Chlebowy, Hood, & LaJoie, 2013; Denham et al., 2007; Peyrot et al., 2015; Rosland et al., 2012; Treadwell et al., 2010). In support of this assertion, our findings expand on the idea that family support may be occurring not just in the present, but may include over time both positive support and barriers between family experiences. Alternatively, as seen in some of our findings, FST suggests that family relationships and multigenerational transmission are not always supportive of prevention or health promotion and in fact may serve as a barrier. To mitigate potential concerns of incorporating family members, it may be pertinent to work in health teams to identify where additional family relationship supports may be beneficial.

It is important for nursing and health professionals to solicit familial experiences with T2DM and current perceptions to gain a more accurate understanding of factors influencing current T2DM-related behaviors. This could provide insight into what role family members play in supporting or inhibiting behavior change. By understanding and acknowledging the communal nature of chronic diseases such as T2DM rather than something only based on individual responsibility, practitioners would be better able to understand an individual’s current state of change as well as build trust with a client who historically may have had less than positive experience with health care practitioners. This research may enable nursing and health care providers to achieve a fuller understanding regarding the familial legacy of T2DM that an African American with a strong history of T2DM may bring to a consultation.

Future research must continue to explore such complexities related to experience with influencing T2DM- related behaviors and beliefs. Research suggests that T2DM programming may benefit from both cultural sensitivity and gender tailoring (Hurt, Seawell, & O’Connor, 2015; Jack, Toston, Jack, & Sims, 2010), although further exploration is needed to better understand the relative risks associated with open or closed family relationships related to T2DM knowledge and behaviors over time. To understand the role that family plays in passing on a T2DM-related legacy, it will also be important for future studies to determine which family members or ages may be most influential. Research may also be warranted to determine whether there are appropriate educational prevention efforts or professional development opportunities for those working with these potentially high-risk T2DM-affected individuals, tailored to specific cultural and gender-based familial experiences (Jack, Toston, Jack, & Sims, 2010).
